# Depression Symptoms as a Mediator Between Long Working Hours and Dental Check‐Ups in Chinese Adults

**DOI:** 10.1155/ijod/4029234

**Published:** 2026-10-01

**Authors:** Ruiying Fu, Yubing Lin, Huang Huang, Peiyuan Sun, Xiuqi Ma, Zhuolun Hu, Yingtai Chen, Xiaofeng Bi, Qiong Chen, Shaokai Zhang, Yin Liu, Ranran Qie, Jiankun Wei, Miaochang Chen, Jieying Zhong, Zhi Qi, Fan Yao, Lijuan Gao, Huanling Yu, Fen Liu, Yao Zhao, Baozhong Chen, Xiaoli Wei, Shasha Qin, Yuhui Du, Guoyu Zhou, Fangfang Yu, Yue Ba, Tingting Shang, Yaqun Zhang, Shan Zheng, Dongmei Xie, Xiaolan Chen, Xiaoling Liu, Cairong Zhu, Weiwei Wu, Yongliang Feng, Ying Wang, Yuting Xie, Qi Yan, Mengyao Wu, Jianhua Gu, Kaiyong Zou, Yawei Zhang

**Affiliations:** ^1^ Department of Cancer Prevention and Control National Cancer Center/National Clinical Research Center for Cancer/Cancer Hospital Chinese Academy of Medical Sciences and Peking Union Medical College Beijing China cacms.ac.cn; ^2^ National Cancer Center/National Clinical Research Center for Cancer/Cancer Hospital Chinese Academy of Medical Sciences and Peking Union Medical College Beijing China cacms.ac.cn; ^3^ Department of Cancer Epidemiology The Affiliated Cancer Hospital of Zhengzhou University and Henan Cancer Hospital Henan Engineering Research Center of Cancer Prevention and Control Henan International Joint Laboratory of Cancer Prevention Zhengzhou Henan China zzu.edu.cn; ^4^ Dongguan Center for Disease Control and Prevention Dongguan Guangdong China; ^5^ Beijing Chaoyang District Center for Disease Control and Prevention Beijing China; ^6^ Department of Nutrition and Food Hygiene School of Public Health Capital Medical University Beijing China ccmu.edu.cn; ^7^ Department of Epidemiology and Health Statistics School of Public Health Capital Medical University Beijing China ccmu.edu.cn; ^8^ Beijing Fangshan District Center for Disease Control and Prevention Beijing China; ^9^ Xi’an Center for Disease Control and Prevention Xi’an Shaanxi China xiancdc.com; ^10^ School of Public Health Zhengzhou University Zhengzhou Henan China zzu.edu.cn; ^11^ Department of Ecology and Environment of Gansu Province Lanzhou Gansu China; ^12^ Lanzhou University Lanzhou Gansu China lzu.edu.cn; ^13^ Genertec Medical Cheng Fei Hospital Chengdu Sichuan China; ^14^ Department of Epidemiology and Health Statistics West China School of Public Health and West China Fourth Hospital Sichuan University Chengdu Sichuan China scu.edu.cn; ^15^ School of Public Health Shanxi Medical University Taiyuan Shanxi China sxmu.edu.cn; ^16^ National Collaborating Centre for Neurological Disorders Prevention Beijing Tiantan Hospital Capital Medical University Beijing China ccmu.edu.cn; ^17^ Department of Emergency Medicine Qilu Hospital of Shandong University Jinan China qiluhospital.com; ^18^ Office for Cancer Screening National Cancer Center/National Clinical Research Center for Cancer/Cancer Hospital Chinese Academy of Medical Sciences and Peking Union Medical College Beijing China cacms.ac.cn

**Keywords:** depression symptoms, long working hours, mediation effect, occupational health, routine dental check-ups

## Abstract

**Aims:**

The relationship between extended working hours and adherence to routine dental check‐ups remains unclear, and whether depressive symptoms act as a potential mediator has yet to be determined. This study aimed to evaluate the association between long working hours and routine dental check‐ups and to assess the mediating effect of depressive symptoms.

**Methods:**

A total of 40,508 participants were included in this cross‐sectional study. Demographic information, psychological factors, frequency of dental check‐ups, and daily working hours were collected through a module‐based electronic questionnaire. Logistic regression models were performed to report the odds ratios (ORs) and 95% confidence intervals (CIs) between long working hours and routine dental check‐ups. Mediation analysis was conducted to assess the mediating role of psychological factors.

**Results:**

Long working hours were inversely associated with routine dental check‐ups among female workers (OR, 95% CI: 0.83, 0.69–0.99), especially female white‐collar workers (0.78, 0.64–0.94), but not male workers (1.05, 0.83–1.32). Long working hours were positively associated with depressive symptoms. Depressive symptoms mediated 6.95% (95% CI 2.09%–34.53%) of the association in females and 5.92% (95% CI 1.92%–18.89%) in female white‐collar workers.

**Conclusion:**

Our study suggested that long working hours affect the psychological status and subsequent utilization of routine dental check‐ups among Chinese female workers. Further longitudinal investigations are warranted to replicate our findings.

## 1. Introduction

Oral diseases contribute substantially to the Global Burden of Disease (GBD). Data from the World Health Organization (WHO) indicated that nearly 3.5 billion individuals were affected by at least one oral disease in 2019 [1]. In China, the prevalence of permanent tooth caries was 21.1% (95% uncertainty interval: 17.7–25.4) based on the GBD for 2023 [2]. Oral diseases can cause toothache, impaired chewing ability, and tooth loss and have also been linked to systemic diseases such as atherosclerosis, diabetes, and cognitive decline [3, 4]. Regular dental attendance is a cornerstone of oral disease prevention and management. Evidence from systematic reviews suggested that regular dental check‐ups were associated with lower caries experience, enhanced self‐rated oral health, and better oral health‐related quality of life [5, 6]. These benefits may be achieved through the prevention and timely treatment of oral diseases, early detection of emerging problems, reinforcement of health behaviors, and improvements in overall well‐being.

In recognition of the importance of preventive dental care, several national health authorities and the World Dental Federation recommend regular dental examinations [7–9]. Many countries have implemented basic oral health insurance, particularly targeting vulnerable groups such as children, adolescents, and older adults. However, working‐age adults are less likely to attend routine dental check‐ups, with only 35% reporting regular visits compared to 43%–47% among other age groups [10, 11]. One possible explanation is the time constraints faced by working individuals, who may prioritize work‐related commitments over preventive health care [12]. From 2016 to 2022, average weekly working hours in China increased steadily, reaching 48.0 h in 2022 [13]. By the end of 2025, this figure had risen further to 48.6 h, indicating that working hours among Chinese employees have remained persistently high [14].

Epidemiological studies from developed countries have shown that long working hours are associated with the postponement or cancelation of routine dental check‐ups [12, 15, 16]. However, time pressure alone does not fully explain this association. Recent evidence indicates that psychological factors may serve as potential mediators affecting dental care utilization among workers [17]. For instance, negative attitudes, dental anxiety, and cognitive barriers have been identified as important psychological factors that reduce the likelihood of attending routine dental check‐ups [18, 19]. These findings imply that long working hours may indirectly affect oral health behaviors by contributing to psychological distress.

Previous research has indicated that prolonged working hours may adversely affect mental health, particularly depression and anxiety [20–24]. In turn, these psychological changes have been linked to reduced utilization of routine dental services, including appointment delays, cancelations, or missed visits [25, 26].

Given the existing evidence, we hypothesize that long working hours negatively influence workers’ psychological well‐being, which subsequently reduces their likelihood of undergoing routine dental check‐ups (Figure 1). To date, limited research has investigated the mediating role of psychological factors in the association between prolonged working hours and routine dental check‐ups. Furthermore, there is limited research on this topic in developing countries such as China, where the average weekly working time reached 48.6 h in the first half of 2024 [27] and only 19.8% of working‐age adults reported undergoing routine dental check‐ups [28]. Given these gaps, we analyzed data from a baseline survey among the Chinese population to examine whether psychological factors partially account for the relationship between prolonged working hours and routine dental check‐ups.

**Figure 1:**
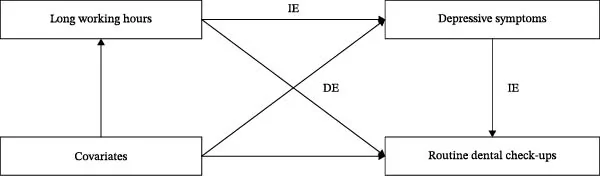
Mediating pathway of the association of long working hours with routine dental check‐ups. Direct acyclic graph of mediation of the association between long working hours with routine dental check‐ups by depressive symptoms. DE, direct effects; IE, indirect effects.

## 2. Methods

### 2.1. Study Population

We conducted a cross‐sectional study using secondary data from a baseline survey of major cancer‐related risk factors among the Chinese population between July 2021 and December 2023. The survey included 116,310 participants, primarily from the provinces of Beijing, Henan, Shanxi, and other regions. After excluding individuals with missing or invalid data on working hours, dental check‐up history, or mental health status, a total of 40,508 (34.8%) individuals were included (Figure S1).

Data were collected using a standardized, module‐based electronic questionnaire through the Smart Health Management Digital Platform for Primary Cancer Prevention (SmartHMDP‐PCP) [29, 30]. The questionnaire captured information on demographics, lifestyle and environmental exposures, occupational history, medical history, and mental health. Electronic informed consent was obtained from all participants prior to their enrollment in the study. The study protocol was reviewed and approved by the Ethics Committee of the National Cancer Center/Cancer Hospital, Chinese Academy of Medical Sciences.

### 2.2. Measures

Long working hours were defined as working more than 11 h per workday in accordance with the International Labor Organization’s criteria [31]. Psychological changes were operationalized as the presence of depressive symptoms [32], assessed using the self‐rate depression scale. Depressive symptoms were defined as a standardized total score of ≥53 [33]. Routine dental check‐ups were defined as undergoing dental care or examination at least once per year as most studies considered a dental visit within 12 months as regular attendance [5].

### 2.3. Covariates

The following covariates were included based on prior literature and potential confounding effects [34–36]. Sociodemographic factors included gender (female/male), age (18–30, 31−39, 40–49, 50−59 years), body mass index (BMI, <18.5, 18.5–23.9, ≥24.0 kg/m^2^), marital status (married/unmarried), education level (below college, college, and above college), and urban–rural residency. Health and behavioral factors included health insurance (yes/no), smoking history (yes/no), drinking history (yes/no), sugary drink and dessert intake (low, moderate, and high), oral disease history (yes/no), and night shift work (yes/no). Smoking history was classified based on lifetime exposure as having smoked more than 100 cigarettes or at least one cigarette daily for ≥1 month. The drinking history was determined by whether participants consumed alcohol at least once weekly for ≥1 month. Sugary drink/dessert consumption was scored by combining sugary drink intake daily (0 = <1 cup, 1 = 1–2 cups, 2 = ≥3 cups) and dessert intake weekly (0 = <1 time, 1 = 1–3 times, 2 = ≥4 times). The total score (range 0–4) was classified as low (0), moderate (1–2), and high (3–4). Based on the International Socioeconomic Index of Occupational Status [37], occupations were categorized as white‐collar (professional, managerial, and clerical roles) and non‐white‐collar (sales, service, agricultural, manual labor, and transport roles).

## 3. Statistical Analysis

Descriptive statistics were computed, with group differences assessed using the chi‐square test. Multivariable logistic regression analyses were employed to evaluate the relationship between long working hours and routine dental check‐ups in the overall population and stratified by sex, age group, and work classification. Association was quantified using odds ratios (ORs) and 95% confidence intervals (CIs). Interaction terms were included in the models to test for effect modification.

To investigate mediation, mediation analysis was performed to decompose the total effect of long working hours on routine dental check‐ups into an average direct effect and an average indirect effect [38]. The proportion attributable to mediation was derived as the indirect effect divided by the total effect [39]. CIs were estimated using a nonparametric Monte Carlo bootstrapping method with 1000 iterations [40]. Missing values were excluded from the regression models.

Three sensitivity analyses were conducted. First, we compared the baseline characteristics between participants included and excluded to evaluate potential selection bias. The second one was the exclusion of participants with a history of oral diseases to focus on preventive dental check‐up behavior. The third one was the exclusion of participants enrolled during the COVID‐19 pandemic to eliminate potential pandemic‐related biases. All statistical analyses were conducted using R Version 4.4.2, and statistical significance was defined as a two‐sided *p*‐value < 0.05.

## 4. Results

Among the 40,508 participants included in the analysis, 67.2% were female workers (Table 1). Overall, 11.2% of participants reported receiving routine dental check‐ups. Compared to those without routine dental utilization, individuals who underwent routine dental utilization were more likely to be female, younger, unmarried, more highly educated, reside in urban areas, have a lower BMI, report no history of smoking or drinking, not engage in night shift work, be white‐collar workers, and report a history of oral diseases. Compared with participants who were excluded, those included in the final analysis had a higher proportion of females, had attained a higher level of education, and lived in urban areas (Table S1).

**Table 1:** Characteristics of the study population by routine dental check‐ups.

Characteristics	Routine dental check‐ups (*N*, %)		Overall (*N* = 40,508)	*p* value ^∗^
	No (*N* = 35,984)	Yes (*N* = 4524)		
Sex				
Female	23,932 (87.9)	3299 (12.1)	27,231 (67.2)	<0.001
Male	12,052 (90.8)	1225 (9.2)	13,277 (32.8)	—
Age group (years)				
18–29	7223 (85.3)	1246 (14.7)	8469 (20.9)	<0.001
30–39	13,440 (88.8)	1688 (11.2)	15,128 (37.3)	—
40–49	10,195 (90.1)	1114 (9.9)	11,309 (27.9)	—
≥50	5126 (91.5)	476 (8.5)	5602 (13.8)	—
Marital status				
Married	28,609 (89.7)	3293 (10.3)	31,902 (78.8)	<0.001
Unmarried	7159 (85.6)	1209 (14.4)	8368 (20.7)	—
Missing	216	22	238 (0.6)	—
Education level				
Below college level	13,111 (90.8)	1335 (9.2)	14,446 (35.7)	<0.001
College	18,398 (87.9)	2536 (12.1)	20,934 (51.7)	—
Above college level	4259 (87.1)	631 (12.9)	4890 (12.1)	—
Missing	216	22	238 (0.6)	—
Urban/rural residency				
Rural	6714 (90.7)	691 (9.3)	7405 (18.3)	<0.001
Urban	28,494 (88.4)	3751 (11.6)	32,245 (79.6)	—
Missing	776	82	858 (2.1)	—
BMI (kg/m^2^)				
<18.5	1616 (83.1)	328 (16.9)	1944 (4.8)	<0.001
18.5–24.0	17,740 (88.0)	2428 (12.0)	20,168 (49.8)	—
≥24.0	16,412 (90.4)	1746 (9.6)	18,158 (44.8)	—
Missing	216	22	238 (0.6)	—
Insurance status				
No	2539 (89.3)	304 (10.7)	2843 (7.0)	0.694
Yes	33,229 (88.8)	4198 (11.2)	37,427 (92.4)	—
Missing	216	22	238 (0.6)	—
Smoking history				
No	30,821 (88.6)	3978 (11.4)	34,799 (85.9)	<0.001
Yes	5151 (90.5)	543 (9.5)	5694 (14.1)	—
Missing	12	3	15 (0.0)	—
Drinking history				
No	29,694 (88.5)	3848 (11.5)	33,542 (82.8)	<0.001
Yes	6286 (90.3)	676 (9.7)	6962 (17.2)	—
Missing	4	0	4 (0.0)	—
Sugary drinks/dessert intake				
Low intake	16,016 (88.9)	1992 (11.1)	18,008 (44.5)	0.427
Moderate intake	18,568 (88.6)	2379 (11.4)	20,947 (51.7)	—
High intake	1372 (90.2)	149 (9.8)	1521 (3.8)	—
Missing	28	4	32 (0.1)	—
Night shift				
No	22,284 (88.2)	2981 (11.8)	25,265 (62.4)	<0.001
Yes	11,621 (89.7)	1331 (10.3)	12,952 (32.0)	—
Missing	2079	212	2291 (5.7)	—
Work classification				
Non‐white‐collar workers	7794 (91.0)	774 (9.0)	8568 (21.2)	<0.001
White‐collar workers	28,014 (88.2)	3740 (11.8)	31,754 (78.4)	—
Missing	176	10	186 (0.5)	—
History of oral disease^a^				
No	19,787 (89.7)	2264 (10.3)	22,051 (54.4)	<0.001
Yes	16,197 (87.8)	2260 (12.2)	18,457 (45.6)	—

^a^Oral diseases included periodontitis, cavities, oral ulcers, and orthodontics.

^∗^Excluded missing value.

In the overall sample, long working hours were not significantly associated with routine dental check‐ups (OR, 95% CI: 0.90, 0.78–1.03), nor were significant associations observed across age groups, job categories, or by the presence of depressive symptoms (Table 2). However, in sex‐stratified analysis, a significant association was observed among female workers (0.83, 0.69–0.99) but not among male workers (1.05, 0.83–1.32).

**Table 2:** Association between long working hours and routine dental check‐ups in the overall population and by sex, age group, work classification, and depressive symptoms.

Long working hours	Regular dental check‐ups (*N*, %)		OR (95% CI)^a^
	No	Yes	
Overall population			
No	33,501 (88.7)	4257 (11.3)	Ref
Yes	2483 (90.3)	267 (9.7)	0.90 (0.78, 1.03)
Sex			
Female			
No	22,459 (87.8)	3133 (12.2)	Ref
Yes	1473 (89.9)	166 (10.1)	0.83 (0.69, 0.99)
Male			
No	11,042 (90.8)	1124 (9.2)	Ref
Yes	1010 (90.9)	101 (9.1)	1.05 (0.83, 1.32)
*p*_interaction_	—	—	0.092
Age group (years)			
18–39			
No	19,077 (87.4)	2740 (12.6)	Ref
Yes	1586 (89.1)	194 (10.9)	0.93 (0.78, 1.09)
≥40			
No	14,424 (90.5)	1517 (9.5)	Ref
Yes	897 (92.5)	73 (7.5)	0.83 (0.63, 1.08)
*p*_interaction_	—	—	0.878
Work classification			
White‐collar workers			
No	26,217 (88.1)	3528 (11.9)	Ref
Yes	1797 (89.4)	212 (10.6)	0.88 (0.75, 1.03)
Non‐white‐collar workers			
No	7121 (90.8)	719 (9.2)	Ref
Yes	673 (92.4)	55 (7.6)	0.99 (0.71, 1.35)
*p*_interaction_	—	—	0.603
Depressive symptoms			
No			
No	24,356 (88.2)	3251 (11.8)	Ref
Yes	1513 (89.8)	171 (10.2)	0.88 (0.73, 1.04)
Yes			
No	9145 (90.1)	1006 (9.9)	Ref
Yes	970 (91.0)	96 (9.0)	0.94 (0.74, 1.19)
*p*_interaction_	—	—	0.548

^a^Adjusted for sex, age group, marital status, education level, urban/rural residency, BMI, insurance status, smoking history, drinking history, sugary drinks/dessert intake, history of oral disease, night shift, work classification, and depressive symptoms.

Further stratification by occupational category revealed that this association was limited to female white‐collar workers, for whom long working hours were significantly associated with a lower likelihood of routine dental check‐ups (0.78, 0.64–0.94, Table 3). Sensitivity analyses excluding participants with a history of oral diseases and those enrolled during the COVID‐19 pandemic yielded results consistent with the main findings (Tables S2–S5). Additionally, among 18–39‐year‐old female workers without oral diseases, a significant association was observed (0.72, 0.53–0.96) (Table S3).

**Table 3:** Association between long working hours and routine dental check‐ups in female population divided by age group, work classification, and depressive symptoms.

Long working hours	Regular dental check‐ups (*N*, %)		OR (95% CI)^a^
	No	Yes	
Age group (years)			
18–39			
No	13,836 (86.6)	2149 (13.4)	Ref
Yes	1060 (89.1)	130 (10.9)	0.84 (0.68, 1.02)
≥40			
No	8623 (89.8)	984 (10.2)	Ref
Yes	413 (92.0)	36 (8.0)	0.78 (0.52, 1.12)
*p*_interaction_	—	—	0.934
Work classification			
White‐collar workers			
No	18,656 (87.5)	2674 (12.5)	Ref
Yes	1194 (89.7)	137 (10.3)	0.78 (0.64, 0.94)
Non‐white‐collar workers			
No	3691 (89.1)	451 (10.9)	Ref
Yes	271 (90.3)	29 (9.7)	1.17 (0.73, 1.82)
*p*_interaction_	—	—	0.058
Depressive symptoms			
No			
No	16,171 (87.2)	2369 (12.8)	Ref
Yes	872 (89.0)	108 (11.0)	0.85 (0.68, 1.05)
Yes			
No	6288 (89.2)	764 (10.8)	Ref
Yes	601 (91.2)	58 (8.8)	0.80 (0.59, 1.06)
*p*_interaction_	—	—	0.798

^a^Adjusted for age group, marital status, education level, urban/rural residency, BMI, insurance status, smoking history, drinking history, sugary drinks/dessert intake, history of oral diseases, night shift, work classification, and depressive symptoms.

Long working hours were significantly associated with higher odds of depressive symptoms in the overall population and persisted across subgroups defined by sex, age, and occupational category (Table 4). These findings were robust in sensitivity analyses (data not shown).

**Table 4:** Association between long working hours and depressive symptoms in overall population and by sex, age group, and work classification.

Long working hours	Depressive symptoms (*N*, %)		OR (95% CI)^a^
	No	Yes	
Overall population			
No	27,607 (73.1)	10,151 (26.9)	Ref
Yes	1684 (61.2)	1066 (38.8)	1.48 (1.35, 1.61)
Sex			
Female			
No	18,540 (72.4)	7052 (27.6)	Ref
Yes	980 (59.8)	659 (40.2)	1.52 (1.36, 1.69)
Male			
No	9067 (74.5)	3099 (25.5)	Ref
Yes	704 (63.4)	407 (36.6)	1.43 (1.24, 1.65)
*p*_interaction_	—	—	0.216
Age group (years)			
18–39			
No	15,331 (70.3)	6486 (29.7)	Ref
Yes	1034 (58.1)	746 (41.9)	1.53 (1.38, 1.70)
≥40			
No	12,276 (77.0)	3665 (23.0)	Ref
Yes	650 (67.0)	320 (33.0)	1.39 (1.19, 1.62)
*p*_interaction_	—	—	0.410
Work classification			
White‐collar workers			
No	22,116 (74.4)	7629 (25.6)	Ref
Yes	1244 (61.9)	765 (38.1)	1.60 (1.45, 1.77)
Non‐white‐collar workers			
No	5380 (68.6)	2460 (31.4)	Ref
Yes	435 (59.8)	293 (40.2)	1.21 (1.02, 1.45)
*p*_interaction_	—	—	0.002

^a^Adjusted for sex, age group, marital status, education level, urban/rural residency, BMI, insurance status, smoking history, drinking history, sugary drinks/dessert intake, history of oral diseases, night shift, and work classification.

Mediation analysis indicated that depressive symptoms accounted for 6.95% (95%CI: 2.09%–34.53%) of the relationship between extended working hours and routine dental check‐ups among all female workers and 5.92% (95%CI: 1.92%–18.89%) among female white‐color workers (Figure 2). These results remained consistent in the sensitivity analyses (Figures S2–S5). Furthermore, a mediating effect of depressive symptoms was also observed among 18–40‐year‐old female workers without oral diseases (Figure S3A).

**Figure 2:**
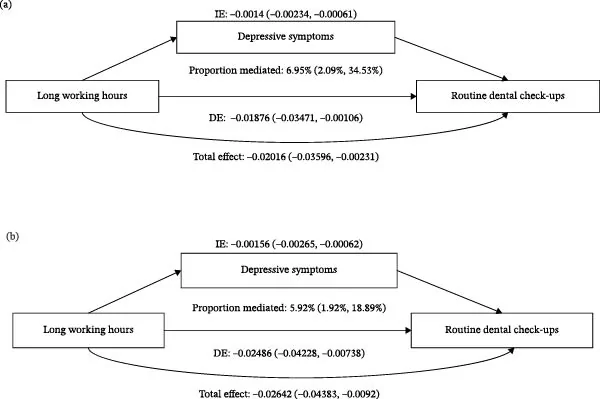
The mediating role of depressive symptoms in females (a) and female white‐collar workers (b). DE, direct effects; IE, indirect effects. Adjusted for age group, marital status, education level, urban/rural residency, BMI, insurance status, smoking history, drinking history, sugary drinks/dessert intake, night shift, and work classification.

## 5. Discussion

To our knowledge, few studies have investigated the relationship between long working hours and routine dental check‐ups in the Chinese population. We found that prolonged working hours were significantly associated with a lower likelihood of routine dental check‐ups among female workers. Furthermore, depressive symptoms partially mediated this relationship, highlighting a potential psychological mechanism linking occupational demands and preventive healthcare behaviors.

Previous studies have indicated that prolonged working hours may be linked with reduced use of dental checkups, although the magnitude and direction of this association vary across populations [12, 15, 16]. Several factors may explain these inconsistencies. First, the prevalence of long working hours differed substantially across countries and populations, ranging from ~6.2% in the United States to 24.95% among men in South Korea. In our study population, the proportion of long working hours was 8.4% among men and 6.0% among women. Second, variation in the definition of routine dental check‐ups might contribute to heterogeneity across studies. While both our study and the South Korean study defined routine care as at least one annual dental visit, the United States study used a biannual measure, and the Japanese study focused specifically on annual preventive dental care.

Third, variations in the accessibility, availability, and affordability of dental services may influence dental care utilization. The distribution of dental healthcare resources is often uneven, with rural areas frequently experiencing shortages of dental professionals and clinics compared with urban regions [41]. In addition, barriers such as travel distance, appointment availability, waiting times, and out‐of‐pocket costs may affect access to preventive dental care. Differences in dental insurance coverage might further influence dental service utilization. For instance, South Korea’s national health insurance covers one annual dental check‐up, whereas routine or preventive dental care is generally not covered in China and Japan [42–44].

Although this study adjusted for several potential confounders, including urban–rural residence and health insurance status, direct measures of dental service accessibility and availability were not available in our dataset. Further studies should incorporate these contextual factors to better understand how healthcare system characteristics influence the association between extended working hours and routine dental check‐up utilization.

Our study also observed a significant relationship between prolonged working hours and depressive symptoms across sex, age, and occupation groups. This is consistent with a meta‐analysis supporting a moderate positive association between prolonged working hours and depressive symptoms in Asian populations [24]. However, earlier studies have reported mixed sex‐specific results. Some studies found this association only in women [45–48], others only in men [35, 49], and still others in both sexes [50–54]. Heterogeneity may arise from differences in the definitions of long working hours and the measurement tools used for depressive symptoms as well as cultural and occupational differences. For example, weekly working hours varied considerably across countries, with Chinese workers averaging 46.1 h per week—higher than those in Japan (36.6 h) and Australia (32.3 h) [13]. Variations in work scheduling (e.g., irregular shifts), which were not assessed in our study, may also contribute to psychological outcomes [49].

Importantly, our study is the first to identify a mediating role of psychological factors in the association between long working hours and routine dental check‐ups, accounting for 6.95% (95% CI: 2.09%–34.53%) of the total association. Previous research has shown that excessive working hours may contribute to the development of psychological distress [23, 24], which in turn reduces the likelihood of engaging in preventive dental care [55, 56]. Several psychological mechanisms might help explain this finding. Depression has been consistently associated with lower frequency of dental visits [26, 57], potentially through cognitive and motivational barriers that reduce both the perceived need for and the ability to seek dental care. In addition, individuals with depressive symptoms may exhibit diminished concern for their personal appearance and oral health, which can lead to delayed or avoided dental checkups [58]. Depression may also impair self‐care motivation more broadly, further contributing to reduced engagement in preventive dental services [59].

The association was observed only among women. In the Chinese sociocultural context, shaped by Confucian values, men are traditionally expected to focus on income generation, while women often bear dual responsibilities for paid work and unpaid domestic labor [60]. According to the role strain theory, individuals juggling multiple roles are more vulnerable to poor physical and mental health. The effort‐recovery model similarly suggests that insufficient recovery time exacerbates psychological stress and fatigue [35]. While the Chinese labor law mandates at least 1 day off per week, this may not be sufficient for women who carry a disproportionate share of household and caregiving responsibilities [61–63], thus limiting their ability to attend dental check‐ups. Additionally, gendered social norms and peer expectations may further influence health behaviors as traditional socialization tends to prioritize men’s needs [64].

Several limitations should be noted. First, all variables were derived from self‐reported data, which may have resulted in a recall bias and exposure misclassification. However, because exposure and outcome information were collected using standardized questionnaires, this misclassification is likely to be nondifferential and could have led to an underestimation of the true association. In addition, compared with excluded participants, those included in the final analysis were characterized by a higher proportion of females and higher educational attainment and resided in urban areas, raising the possibility of selection bias. Because these characteristics are also associated with healthcare‐seeking behaviors, the observed associations may have been either underestimated or overestimated. To mitigate this concern, we adjusted for sex, educational attainment, residential area, and other relevant covariates in the multivariate analyses. Nevertheless, residual selection bias cannot be completely excluded, and the extent to which findings can be extrapolated to populations with different demographic characteristics remains uncertain. Further studies with more representative populations are needed to confirm our findings.

Second, the cross‐sectional design prevented us from establishing temporal or causal inference. Third, although the study included multiple provinces and a large sample size, generalizability to the broader Chinese population may be limited by the reliance on smartphone‐based data collection. Nevertheless, smartphone penetration in China was about 72% in 2022 [65], suggesting reasonable representativeness. Fourth, several subgroup analyses were conducted to examine potential heterogeneity by sex, age, work classification, and depressive symptom status. We acknowledge that multiple testing may increase the likelihood of type I error and that some subgroup findings may have occurred by chance. These analyses were hypothesis‐driven and intended to explore potential effect modification rather than to provide a definitive causal inference. Accordingly, results should be interpreted in light of their consistency, effect size, and biological or psychosocial plausibility rather than solely on the statistical significance. Future studies with prespecified hypotheses and adequate power are needed to confirm these subgroup‐specific associations.

## 6. Conclusion

This study provides novel evidence that long working hours may contribute to the postponement or cancelation of routine dental checkups among Chinese women, particularly white‐collar workers. The mediating effect of depressive symptoms in this association highlights a potential psychological pathway linking occupational stress to preventive health behavior. These findings emphasize the value of workplace strategies and mental health interventions to support access to preventive dental care, especially among working women.

## Author Contributions

Yawei Zhang designed and supervised the research. Yawei Zhang and Ruiying Fu developed the study methodology and analytical framework. Huang Huang, Peiyuan Sun, Xiuqi Ma, Zhuolun Hu, Yingtai Chen, Xiaofeng Bi, Qiong Chen, Shaokai Zhang, Yin Liu, Jiankun Wei, Miaochang Chen, Jieying Zhong, Zhi Qi, Fan Yao, Lijuan Gao, Huanling Yu, Fen Liu, Yao Zhao, Baozhong Chen, Xiaoli Wei, Shasha Qin, Yuhui Du, Guoyu Zhou, Fangfang Yu, Yue Ba, Tingting Shang, Yaqun Zhang, Shan Zheng, Dongmei Xie, Xiaolan Chen, Xiaoling Liu, Cairong Zhu, Weiwei Wu, Yongliang Feng, Ying Wang, Kaiyong Zou, and Yawei Zhang contributed to data collection. Yuting Xie, Ranran Qie, Qi Yan, Mengyao Wu, Peiyuan Sun, Ruiying Fu, and Yubing Lin cleaned the data. Ruiying Fu and Yubing Lin performed data analysis. Ruiying Fu and Jianhua Gu performed the data graphic. Ruiying Fu wrote the manuscript. Yawei Zhang revised the paper critically for important content.

## Funding

This study was supported by the Capital’s Funds for Health Improvement and Research (Grant 2022‐1G‐4023), the National Natural Science Foundation of China (Grant 82204168), and the Non‐profit Central Research Institute Fund of Chinese Academy of Medical Sciences (Grant 2021‐RC310‐009).

## Disclosure

All authors have read and approved the final version of the manuscript. Yawei Zhang had full access to all of the data in this study and takes complete responsibility for the integrity of the data and the accuracy of the data analysis.

## Ethics Statement

All study procedures were approved by the Ethics Committee of the National Cancer Center/Cancer Hospital, Chinese Academy of Medical Sciences. The procedures used in this study adhere to the tenets of the Declaration of Helsinki.

## Consent

Informed consent was obtained from all individual participants involved in the study.

## Conflicts of Interest

The authors declare no conflicts of interest.

## Supporting Information

Additional supporting information can be found online in the Supporting Information section.

## Supporting information


**Supporting Information** Table S1: Characteristics of the excluded and included participants. Table S2: Association between long working hours and routine dental check‐ups in the overall population and by sex without a history of oral diseases. Table S3: Association between long working hours and routine dental check‐ups in females without history of oral diseases divided by age group, work classification, and depressive symptoms. Table S4: Association between long working hours and routine dental check‐ups in the overall population and subgroup population divided by sex after COVID‐19. Table S5: The association between long working hours and routine dental check‐ups in females was divided by age group, work classification, and depressive symptoms after COVID‐19. Figure S1: Flowchart of the study population. Figure S2: The mediating role of depressive symptoms in female workers without a history of oral diseases. Figure S3: The mediating role of depressive symptoms in 18–39‐year‐old females and in female white‐collar workers without oral diseases. (a) The mediating role of depressive symptoms in 18–39‐year‐old females without oral diseases. (b) The mediating role of depressive symptoms in female white‐collar workers without oral diseases. Figure S4: The mediating role of depressive symptoms in females after COVID‐19. Figure S5: The mediating role of depressive symptoms in female white‐collar workers after COVID‐19.

## Data Availability

The data that support the findings of this study are available from the corresponding author upon reasonable request.
