# Supplementary material for: Depression Symptoms as a Mediator Between Long Working Hours and Dental Check‐Ups in Chinese Adults
Source: Int J Dent. 2026 Oct 1;2026:4029234. doi: 10.1155/ijod/4029234 (PMC13628571; doi:10.1155/ijod/4029234)
Supplement: Supplementary file 1 [file IJOD-2026-4029234-s001.docx]

**Supplementary material**

[Supplementary Table 1. Characteristics of the excluded and included participants.	2](#_Toc243)

[Supplementary Table 2. Association between long working hours and routine dental check-ups in the overall population and by sex without a history of oral diseases.	4](#_Toc8722)

[Supplementary Table 3 Association between long working hours and routine dental check-ups in females without history of oral diseases divided by age group, work classification, and depressive symptoms.	5](#_Toc7754)

[Supplementary Table 4 Association between long working hours and routine dental check-ups in the overall population and subgroup population divided by sex after COVID-19.	6](#_Toc9237)

[Supplementary Table 5 The association between long working hours and routine dental check-ups in females was divided by age group, work classification, and depressive symptoms after COVID-19.	7](#_Toc6094)

[Supplementary Fig. 1 Flowchart of the study population.	8](#_Toc4105)

[Supplementary Fig. 2 The mediating role of depressive symptoms in female workers without a history of oral diseases.	9](#_Toc17688)

[Supplementary Fig. 3 The mediating role of depressive symptoms in 18-39-year-old females (Supplementary Fig. 3a) and in female white-collar workers (Supplementary Fig. 3b) without oral diseases.	10](#_Toc17625)

[Supplementary Fig. 4 The mediating role of depressive symptoms in females after COVID-19.	11](#_Toc16441)

[Supplementary Fig. 5 The mediating role of depressive symptoms in female white-collar workers after COVID-19.	12](#_Toc6114)

# Supplementary Table 1. Characteristics of the excluded and included participants.

|  | Participants (N, %) | | Overall (N=116,310) | *P* value ^*^ |
| --- | --- | --- | --- | --- |
|  | Exclusion (N=75,802) | Inclusion (N=40,508) |  |  |
| **Sex** |  |  |  |  |
| Female | 45,510 (60.0) | 27,231 (67.2) | 72,741 (62.5) | <0.001 |
| Male | 30,292 (40.0) | 13,277 (32.8) | 43,569 (37.5) |  |
| **Age group (years)** |  |  |  |  |
| 18-29 | 20,172 (26.6) | 8,469 (20.9) | 28,641 (24.6) | <0.001 |
| 30-39 | 17,253 (22.8) | 15,128 (37.3) | 32,381 (27.8) |  |
| 40-49 | 16,063 (21.2) | 11,309 (27.9) | 27,372 (23.5) |  |
| ≥50 | 22,314 (29.4) | 5,602 (13.8) | 27,916 (24.0) |  |
| **Marital status** |  |  |  |  |
| Married | 48,287 (63.7) | 31,902 (78.8) | 80,189 (68.9) | <0.001 |
| Unmarried | 18,168 (24.0) | 8,368 (20.7) | 26,536 (22.8) |  |
| Missing | 9,347 (12.3) | 238 (0.6) | 9,585 (8.2) |  |
| **Education level** |  |  |  |  |
| Below college level | 39,288 (51.8) | 14,446 (35.7) | 53,734 (46.2) | <0.001 |
| College | 21,504 (28.4) | 20,934 (51.7) | 42,438 (36.5) |  |
| Above college level | 5,663 (7.5) | 4,890 (12.1) | 10,553 (9.1) |  |
| Missing | 9,347 (12.3) | 238 (0.6) | 9,585 (8.2) |  |
| **Urban/rural residency** |  |  |  |  |
| Rural | 20,774 (27.4) | 7,405 (18.3) | 28,179 (24.2) | <0.001 |
| Urban | 46,894 (61.9) | 32,245 (79.6) | 79,139 (68.0) |  |
| Missing | 8,134 (10.7) | 858 (2.1) | 8,992 (7.7) |  |
| **BMI, Kg/m^2^** |  |  |  |  |
| <18.5 | 3,598 (4.7) | 1,944 (4.8) | 5,542 (4.8) | <0.001 |
| 18.5-24.0 | 32,654 (43.1) | 20,168 (49.8) | 52,822 (45.4) |  |
| ≥24.0 | 30,203 (39.8) | 18,158 (44.8) | 48,361 (41.6) |  |
| Missing | 9,347 (12.3) | 238 (0.6) | 9,585 (8.2) |  |
| **Insurance status** |  |  |  |  |
| No | 11,041 (14.6) | 2843 (7.0) | 13,884 (11.9) | <0.001 |
| Yes | 55,414 (73.1) | 37,427 (92.4) | 92,841 (79.8) |  |
| Missing | 9,347 (12.3) | 238 (0.6) | 9,585 (8.2) |  |
| **Smoking history** |  |  |  |  |
| No | 50,916 (67.2) | 34,799 (85.9) | 85,715 (73.7) | <0.001 |
| Yes | 9,130 (12.0) | 5,694 (14.1) | 14,824 (12.7) |  |
| Missing | 15,756 (20.8) | 15 (0.0) | 15,771 (13.6) |  |
| **Drinking history** |  |  |  |  |
| No | 49,735 (65.6) | 33,542 (82.8) | 83,277 (71.6) | <0.001 |
| Yes | 8,666 (11.4) | 6,962 (17.2) | 15,628 (13.4) |  |
| Missing | 17,401 (23.0) | 4 (0.0) | 17,405 (15.0) |  |
| **Sugary drinks/dessert intake** |  |  |  |  |
| Low intake | 29,070 (38.4) | 18,008 (44.5) | 47,078 (40.5) | <0.001 |
| Moderate intake | 21,400 (28.2) | 20,947 (51.7) | 42,347 (36.4) |  |
| High intake | 1,956 (2.6) | 1,521 (3.8) | 3,477 (3.0) |  |
| Missing | 23,376 (30.8) | 32 (0.1) | 23,408 (20.1) |  |
| **Night shift** |  |  |  |  |
| No | 10,075 (13.3) | 25,265 (62.4) | 35,340 (30.4) | <0.001 |
| Yes | 10,611 (14.0) | 12,952 (32.0) | 23,563 (20.3) |  |
| Missing | 55,116 (72.7) | 2,291 (5.7) | 57,407 (49.4) |  |
| **Work classification** |  |  |  |  |
| Non-white-collar workers | 5,276 (7.0) | 8,568 (21.2) | 13,844 (11.9) | <0.001 |
| White-collar workers | 13,867 (18.3) | 31,754 (78.4) | 45,621 (39.2) |  |
| Missing | 56,659 (74.7) | 186 (0.5) | 56,845 (48.9) |  |
| **History of oral disease** ^a^ |  |  |  |  |
| No | 64,680 (85.3) | 22,051 (54.4) | 86,731 (74.6) | <0.001 |
| Yes | 11,122 (14.7) | 18,457 (45.6) | 29,579 (25.4) |  |
| **Long working hours** |  |  |  |  |
| No | 17,733 (23.4) | 37,758 (93.2) | 55,491 (47.7) | <0.001 |
| Yes | 1,559 (2.1) | 2,750 (6.8) | 4,309 (3.7) |  |
| Missing | 56,510 (74.5) | 0 (0) | 56,510 (48.6) |  |
| **Regular dental check-ups** |  |  |  |  |
| No | 41,411 (54.6) | 35,984 (88.8) | 77,395 (66.5) | <0.001 |
| Yes | 3,564 (4.7) | 4,524 (11.2) | 8,088 (7.0) |  |
| Missing | 30,827 (40.7) | 0 (0) | 30,827 (26.5) |  |

^*^ Excluded missing value.

^a^ Oral diseases included periodontitis, cavities, oral ulcers, and orthodontics.

# Supplementary Table 2. Association between long working hours and routine dental check-ups in the overall population and by sex without a history of oral diseases.

| Long working hours | Routine dental check-ups (N, %) | | OR (95% CI) ^*^ |
| --- | --- | --- | --- |
|  | No | Yes |  |
| **Overall population** |  |  |  |
| No | 18,353 (89.6) | 2,130 (10.4) | Ref |
| Yes | 1,421 (91.4) | 133 (8.6) | 0.87 (0.71, 1.06) |
| **Sex** |  |  |  |
| **Female** |  |  |  |
| No | 11,934 (88.8) | 1,508 (11.2) | Ref |
| Yes | 824 (91.6) | 76 (8.4) | 0.76 (0.58, 0.97) |
| **Male** |  |  |  |
| No | 6,419 (91.2) | 622 (8.8) | Ref |
| Yes | 597 (91.3) | 57 (8.7) | 1.09 (0.79, 1.47) |
| P _interaction_ |  |  | 0.073 |

^*^ Adjusted for sex, age group, marital status, education level, urban/rural residency, BMI, insurance status, smoking history, drinking history, sugary drinks/dessert intake, night shift, work classification, and depressive symptoms.

# Supplementary Table 3 Association between long working hours and routine dental check-ups in females without history of oral diseases divided by age group, work classification, and depressive symptoms.

| Long working hours | Routine dental check-ups (N, %) | | OR (95% CI) ^*^ |
| --- | --- | --- | --- |
|  | No | Yes |  |
| **Age group (years)** |  |  |  |
| **18-39** |  |  |  |
| No | 7,501 (87.7) | 1,052 (12.3) | Ref |
| Yes | 602 (91.6) | 55 (8.4) | 0.72 (0.53, 0.96) |
| **≥40** |  |  |  |
| No | 4,441 (90.7) | 456 (9.3) | Ref |
| Yes | 224 (91.4) | 21 (8.6) | 0.85 (0.49, 1.37) |
| P _interaction_ |  |  | 0.255 |
| **Work classification** |  |  |  |
| **White-collar workers** |  |  |  |
| No | 9,764 (88.5) | 1,268 (11.5) | Ref |
| Yes | 651 (91.7) | 59 (8.3) | 0.68 (0.50, 0.90) |
| **Non-white-collar workers** |  |  |  |
| No | 2,109 (89.9) | 237 (10.1) | Ref |
| Yes | 170 (90.9) | 17 (9.1) | 1.14 (0.62, 2.00) |
| P _interaction_ |  |  | 0.051 |
| **Depressive symptoms** |  |  |  |
| **No** |  |  |  |
| No | 8,517 (88.2) | 1,143 (11.8) | Ref |
| Yes | 494 (91.0) | 49 (9.0) | 0.74 (0.53, 1.01) |
| **Yes** |  |  |  |
| No | 3,425 (90.4) | 365 (9.6) | Ref |
| Yes | 332 (92.5) | 27 (7.5) | 0.80 (0.51, 1.19) |
| P _interaction_ |  |  | 0.628 |

^*^ Adjusted for age group, marital status, education level, urban/rural residency, BMI, insurance status, smoking history, drinking history, sugary drinks/dessert intake, night shift, work classification, and depressive symptoms.

# Supplementary Table 4 Association between long working hours and routine dental check-ups in the overall population and subgroup population divided by sex after COVID-19.

| **Long working hours** | **Routine dental check-ups (N, %)** | | **OR (95% CI)** ^*^ |
| --- | --- | --- | --- |
|  | **No** | **Yes** |  |
| **Overall population** |  |  |  |
| No | 32205 (88.8) | 4064 (11.2) | Ref |
| Yes | 2402 (90.4) | 256 (9.6) | 0.89 (0.77, 1.03) |
| **Sex** |  |  |  |
| **Female** |  |  |  |
| No | 21657 (87.8) | 2998 (12.2) | Ref |
| Yes | 1448 (90.1) | 159 (9.9) | 0.81 (0.68, 0.97) |
| **Male** |  |  |  |
| No | 10548 (90.8) | 1066 (9.2) | Ref |
| Yes | 954 (90.8) | 97 (9.2) | 1.06 (0.83, 1.34) |
| P _interaction_ |  |  | 0.055 |

^*^ Adjusted for sex, age group, marital status, education level, urban/rural residency, BMI, insurance status, smoking history, sugary drinks/dessert intake, history of oral diseases, night shift, work classification, and depressive symptoms.

# Supplementary Table 5 The association between long working hours and routine dental check-ups in females was divided by age group, work classification, and depressive symptoms after COVID-19.

| Long working hours | Routine dental check-ups (N, %) | | OR (95% CI) ^*^ |
| --- | --- | --- | --- |
|  | No | Yes |  |
| **Age group (years)** |  |  |  |
| **18-39** |  |  |  |
| No | 13,450 (86.7) | 2,066 (13.3) | Ref |
| Yes | 1,046 (89.2) | 127 (10.8) | 0.83 (0.68, 1.01) |
| **≥40** |  |  |  |
| No | 8,207 (89.8) | 932 (10.2) | Ref |
| Yes | 402 (92.6) | 32 (7.4) | 0.72 (0.47, 1.06) |
| P _interaction_ |  |  | 0.796 |
| **Work classification** |  |  |  |
| **White-collar workers** |  |  |  |
| No | 18,076 (87.6) | 2,566 (12.4) | Ref |
| Yes | 1,185 (90.0) | 132 (10.0) | 0.77 (0.63, 0.93) |
| **Non-white-collar workers** |  |  |  |
| No | 3,474 (89.1) | 424 (10.9) | Ref |
| Yes | 255 (90.4) | 27 (9.6) | 1.09 (0.67, 1.72) |
| P _interaction_ |  |  | 0.100 |
| **Depressive symptoms** |  |  |  |
| **No** |  |  |  |
| No | 15,609 (87.3) | 2,261 (12.7) | Ref |
| Yes | 856 (89.2) | 104 (10.8) | 0.82 (0.66, 1.03) |
| **Yes** |  |  |  |
| No | 6,048 (89.1) | 737 (10.9) | Ref |
| Yes | 592 (91.5) | 55 (8.5) | 0.79 (0.58, 1.05) |
| P _interaction_ |  |  | 0.869 |

^*^ Adjusted for age, marital status, education level, urban/rural residency, BMI, insurance status, smoking history, sugary drinks/dessert intake, history of oral diseases, night shift, work classification, and depressive symptoms.

# Supplementary Fig. 1 Flowchart of the study population.

# Supplementary Fig. 2 The mediating role of depressive symptoms in female workers without a history of oral diseases.

**IE** indicates indirect effect; **DE**, direct effect.

Adjusted for age group, marital status, education level, urban/rural residency, BMI, insurance status, smoking history, drinking history, sugary drinks/dessert intake, night shift, and work classification.

# Supplementary Fig. 3 The mediating role of depressive symptoms in 18-39-year-old females (Supplementary Fig. 3a) and in female white-collar workers (Supplementary Fig. 3b) without oral diseases.

**IE** indicates indirect effect; **DE**, direct effect.

Adjusted for age group, marital status, education level, urban/rural residency, BMI, insurance status, smoking history, drinking history, sugary drinks/dessert intake, night shift, and work classification.

# Supplementary Fig. 4 The mediating role of depressive symptoms in females after COVID-19.

**IE** indicates indirect effect; **DE**, direct effect.

Adjusted for age group, marital status, education level, urban/rural residency, BMI, insurance status, smoking history, drinking history, sugary drinks/dessert intake, history of oral diseases, night shift, and work classification.

# Supplementary Fig. 5 The mediating role of depressive symptoms in female white-collar workers after COVID-19.

**IE** indicates indirect effect; **DE**, direct effect.

Adjusted for age, marital status, education level, urban/rural residency, BMI, insurance status, smoking history, drinking history, sugary drinks/dessert intake, history of oral diseases, and night shift.
